# Evaluation of redox profiles in exogenous subclinical hyperthyroidism at two different levels of TSH suppression

**DOI:** 10.20945/2359-3997000000075

**Published:** 2018-10-01

**Authors:** Bruna Karoline Lima Piazera, Diego Viana Gomes, Patrícia Vigário, Verônica P. Salerno, Mário Vaisman

**Affiliations:** 1 Universidade Federal do Rio de Janeiro Universidade Federal do Rio de Janeiro Departamento de Endocrinologia Rio de Janeiro RJ Brasil Departamento de Endocrinologia, Universidade Federal do Rio de Janeiro (UFRJ), Rio de Janeiro, RJ, Brasil; 2 Universidade Federal do Rio de Janeiro Universidade Federal do Rio de Janeiro Escola de Educação Física e Desporto (EEFD) Laboratório de Bioquímica do Exercício e Motores Moleculares (LaBEMMol) Rio de Janeiro RJ Brasil Laboratório de Bioquímica do Exercício e Motores Moleculares (LaBEMMol), Escola de Educação Física e Desporto (EEFD), Universidade Federal do Rio de Janeiro (UFRJ), Rio de Janeiro, RJ, Brasil; 3 Centro Universitário Augusto Motta Centro Universitário Augusto Motta Programa de Pós-Graduação em Ciências da Reabilitação Rio de Janeiro RJ Brasil Programa de Pós-Graduação em Ciências da Reabilitação, Centro Universitário Augusto Motta (Unisuam), Rio de Janeiro, RJ, Brasil

**Keywords:** Subclinical hyperthyroidism, thyroidectomy, oxidative stress, antioxidants

## Abstract

**Objective:**

Evaluate the relationship between exogenous subclinical hyperthyroidism and oxidative stress through the analysis of the redox profile of patients with subclinical hyperthyroidism exogenous (SCH) grade I (TSH = 0.1 to 0.4 IU/mL) and grade II (TSH < 0.1 IU/mL).

**Subjects and methods:**

We analyzed 46 patients with SCH due to the use of TSH suppressive therapy with LT4 after total thyroidectomy along with 6 control euthyroid individuals (3M and 3W). Patients were divided into two groups, G1 with TSH ≥ 0.1-0.4 IU/mL (n = 25; and 7M 14W) and G2 with TSH < 0.1 IU/mL (n = 25; and 4M 21W). Venous blood samples were collected to measure the levels of markers for oxidative damage (TBARS, FOX and protein carbonylation), muscle and liver damage (CK, AST, ALT, GGT) and antioxidants (GSH, GSSG and catalase).

**Results:**

Individuals in G2 showed a GSH/GSSG ratio ~ 30% greater than those in G1 (p = 0.004) and a catalase activity that was 4 times higher (p = 0.005). For lipid peroxidation, the levels measured in G2 were higher than both control and G1 (p = 0.05). No differences were observed for both protein carbonyl markers. G1 and G2 presented with greater indications of cell injury markers than the control group.

**Conclusion:**

TSH suppression therapy with LT4 that results in subclinical hyperthyroidism can cause a redox imbalance. The greater antioxidant capacity observed in the more suppressed group was not sufficient to avoid lipid peroxidation and cellular damage.

## INTRODUCTION

Thyroid cancer is the most common malignancy to affect the endocrine system. Its incidence has increased worldwide in recent years with differentiated thyroid carcinoma (DTC) accounting for close to 90% of all cases (1). Treatments for DTC currently involve a partial or total thyroidectomy followed by radioiodine ablation and hormone suppression with levothyroxine (LT4) (2). The suppression of thyroid stimulating hormone (TSH) using LT4 at above physiological doses is widely used to reduce tumor recurrence, which induces exogenous subclinical hyperthyroidism (SCH) (3).

SCH is characterized by normal levels of thyroid hormones associated with low or undetectable TSH values (4). Studies have demonstrated that SCH has similar systemic effects to clinical hyperthyroidism that includes an increased metabolism resulting in a greater production of free radicals and alterations in redox balance leading to tissue damage (5–7). An imbalance between levels in free radicals and antioxidants is associated with some diseases, like diabetes and arterial coronary disease (8). Coronary disease is also associated with subclinical hyperthyroidism, because it leads to impaired endothelial function, oxidative stress and decreased insulin sensitivity (8–10).

An understanding of the effects associated with diseases and medications are important to prevent and treat possible collateral damage (11). Previous studies on oxidative stress in endogenous SCH have shown that there is an increase in lipid peroxidation and in the activity of antioxidant enzymes, such as SOD (5,12,13). However, until now, the oxidative profile of patients with exogenous SCH due to TSH suppression with LT4 as a treatment for DTC is not well known (8), as well as the effect of different levels of TSH suppression on the oxidative profile.

Therefore, the aim of this study was to evaluate the redox status of euthyroid patients with exogenous SCH, comparing them according to two categories of TSH suppression: ≥ 0.1 to 0.4 IU/mL (grade 1) and < 0.1 IU/mL (grade 2) to the control group (euthyroid). The knowledge that the level of TSH suppression may influence the oxidative profile of SCH patients can be useful for making clinical decisions, including adjustments in the TSH levels that could be associated with an improved general health status.

## SUBJECTS AND METHODS

### Study and participants

A cross-sectional study was conducted. First, 74 patients were recruited from the Endocrine Clinic of the *Hospital Clementino Fraga Filho* (HUCFF, Rio de Janeiro, Brazil) that consisted of 6 euthyroid control individuals and 68 patients with exogenous SCH due to TSH suppressive therapy with LT4 after total thyroidectomy for DTC. All patients were 18 years of age or older and exhibited a stable health profile over the last year of follow-up exams. After a clinical examination, 22 subjects were excluded due to characteristics that could affect the oxidative stress measurements including: detectable thyroglobulin, positive whole body scan (PCI), C-reactive protein greater than 3.0 ng/L, possible current malignancy or serious illnesses, smokers, alcoholics and those on medications that could influence the oxidative stress.

None of the 46 SCH (34 women and 12 men) patients included in the study had active thyroid tissue and all had at least six months of suppressive therapy with LT4. Each had at least two dosages with TSH below the reference value (< 0.4 IU/mL) and normal FT4 (0.8 to 1.9 ng/dL) with undetectable thyroglobulin, anti-thyroglobulin antibody negative and negative whole-body scans.

This study was approved by the local ethics committee (040/11-CEP) and all patients gave their written consent before the study entry.

### Procedures

The participants were divided according to the TSH value into three groups, as proposed by Biondi and cols. (2015): Control: 0.4 to 4 IU/ml; G1: TSH ≥ 0.1 to 0.4 IU/mL (n = 21) and G2: TSH < 0.1 IU/mL (n = 25). Fasting venous blood samples were collected at rest. Liver and muscle enzymes were measured in plasma because lipid peroxidation changes the membrane lipids and can lead to leakage of the cytosolic enzymes to the plasma (14,15).

The samples were centrifuged (1500 x g ≅ 4°C, 15 min) for plasma separation. Aliquots of 1 mL plasma were separated and stored at −80°C until analysis. After the plasma was removed, the erythrocytes were resuspended 1:1 in 0.9% saline and centrifuged (1500 X g ≅ 4°C, 15 min). The supernatant was discarded and the pellet was resuspended again at the same proportion until the process was performed three times.

The following hormones and biomarkers were considered in the study:

Thyroid-stimulating hormone - TSH: TSH was measured with a chemiluminescent immunometric assay third generation commercial kit using DPC^®^ (Diagnostic Products Corporation, USA), following the manufacturer's instructions. The reading was performed in the Immulite 2000 system. The reference value for TSH was 0.4 to 4.0 IU/mL with an intraassay variation of 3.8%-12.5% and inter-assay of 4.6%- 12.5%.

Free thyroxine – FT4: Free T4 was measured by a chemiluminescent immunoenzymatic assay using the DPC^®^ commercial kit (Diagnostic Products Corporation, USA), according to the manufacturer's instructions. The reading was performed in automatic device (Immulite 2000^®^). The reference value for T4 was 0.8-1.9 ng/dL with an intra-assay variation of 4.4%-7.5% and inter-assay variation of 4.8%-9.0%.

### Biomarkers of cellular injury to liver and muscle

The plasma levels of the enzymes aspartate aminotransferase (AST), alanine aminotransferase (ALT), gamma glutamyl transferase (GGT) and creatine kinase (CK) were measured using commercial kits (Bioclin^®^, Brazil) following the manufacturer's specifications. Controls included the use of two control sera along with a Biocontrol normal and pathological (Bioclin^®^, Brazil).

### Thiobarbituric acid reactive substances – TBARs

To determine plasma levels of TBARs, 50 μL of plasma was diluted with 150 μL of 100 mM phosphate buffer. Next, an equal volume (200 μL) of 10% Trichloroacetic acid (TCA) was added, mixed and allowed to incubate for 15 min at 4°C before centrifugation (2200 x g for 15 minutes). To the supernatant (300 μL), an equal volume of TBA (465 mM thiobarbituric acid in 0.1 M Dimethyl Sulfoxide - DMSO) was added and the mixture heated at 95°C for 2 hours. After 15 min of cooling to room temperature, the absorbance of samples (200 μL) were measured at 532 nm in an ELISA plate. The concentration was calculated by a standard curve of TMP and the values presented in nmol MDA (16).

### Ferrous oxidation xylenol orange - FOX

To 25 μL plasma sample, 25 μL of 40 mM butylated hydroxytoluene (BHT; 45 mg in 5 mL of 100% methanol) was added. The samples were vortexed for 1 minute and then centrifuged (5000 x g, 5 min ≅ 4°C). To the supernatant (20 μL), 360 μL of the working reagent (100 mM orange xylenol, 25 mM sulfuric acid and 250 mM ferrous sulfate diluted in 90% methanol) was added. The mixture was incubated at room temperature protected from light. After 2h, samples were centrifuged (10,000 x g, 10 min ≅ 4°C). The absorbance of the supernatant (200 μL) was read in an Elisa plate 560 nm. The lipid hydroperoxide concentration was calculated using the molar extinction coefficient of 4.46 × 10^4^ M^−1^cm^−1^. The values are presented as nmol/L (17–19).

### Protein carbonylation

To measure protein carbonylation, plasma (200 μL) was treated with 500 μL of 10 mM 2,4- dinitrophenylhydrazine (DNPH). A control blank was prepared at the same time for each sample. Next, 200 μL of the sample was mixed with 500 μL of HCl (2.5 M) followed by an incubation for one hour in the dark with vortexing every 15 minutes. This was followed by the addition of 1 mL of 10% TCA (10%) and centrifugation (15,000 x g 5 min ≅ 4°C). The supernatant was discarded and the prellet was washed with 1 mL of a 1:1 (v/v) mixture of ethyl acetate and ethanol 1:1. This was repeated three times. After the last wash, the pellet was resuspended in 1 mL of urea (6 M) and incubated at 37°C with shaking for 15 minutes. The absorbance was measured at 375 nm, and the data were calculated by molar extinction coefficient and expressed as mmol/mg protein (20).

### Reduced gluthatione/gluthatione oxidized - GSH/GSSG ratio

GSH and GSSG were evaluated to determine if there was an upregulation of the antioxidant system that could explain the absence of cell damage. Assays for reduced gluthatione (GSH) and gluthatione oxidized (GSSG) were performed as described by Rahman and cols. (21). Briefly, 70 μL of sulfosalicylic acid (0.23 M) was added to plasma (200 μL). The mixture was immediately centrifuged (15,000 x g for 10 min ≅ 4°C). The supernatant was removed, aliquoted and stored at −80°C until the time of analysis.

To perform GSH measurements, 1.4 mL of buffer (100 mM potassium phosphate and 5 mM EDTA) was added to 200 μL of plasma. Then, 120 μL of 1.7 mM 5,5-Dithiobis (2-nitrobenzoic acid) (DTNB) and 120 μL glutathione reductase (500 units) were added. The mixture was homogenized for thirty seconds before the addition of 120 μL of 0.8 mM β-nicotinamide adenine dinucleotide 2’-phosphate (β-NADPH). The absorbance at 420 nm was measured for 120 seconds every thirty seconds. The concentration was expressed in μM based on the change in absorbance generated by the formation of 2-nitro-5- thiobenzoic. The final concentration was obtained from a linear regression of a GSH standard curve.

Oxidized glutathione (GSSG) was measured by adding 2 μL of vinylpyridine to 200 μL of plasma followed by a 1h incubation in the dark. Next, 12 μL of triethanolamine was added to adjust the pH. The subsequent steps were identical to the GSH assay.

### Catalase

Enzyme activity was measured in erythrocytes as described by Aebi (22). Briefly, 10 μL of erythrocytes were diluted 200-fold and analyzed in 1 mL of a solution containing 50 mM PBS (pH 7.0) in the absence or presence of 30 mM H_2_O_2_. Absorbance was measured at 240 nm for 90 seconds. The absorbance of the samples and blank were recorded every 15 seconds. The results were expressed as k/mg Hb.

### Statistics

All data were expressed as mean ± standard deviation. The comparisons between the two groups of differing TSH suppression levels were performed with the Kruskal-Wallis test and a Dunn's test for multiple comparisons. All analyses were made using GraphPad Prism software, version 6. Statistical significance was defined by a p < 0.05.

## RESULTS

**Figure 1:**
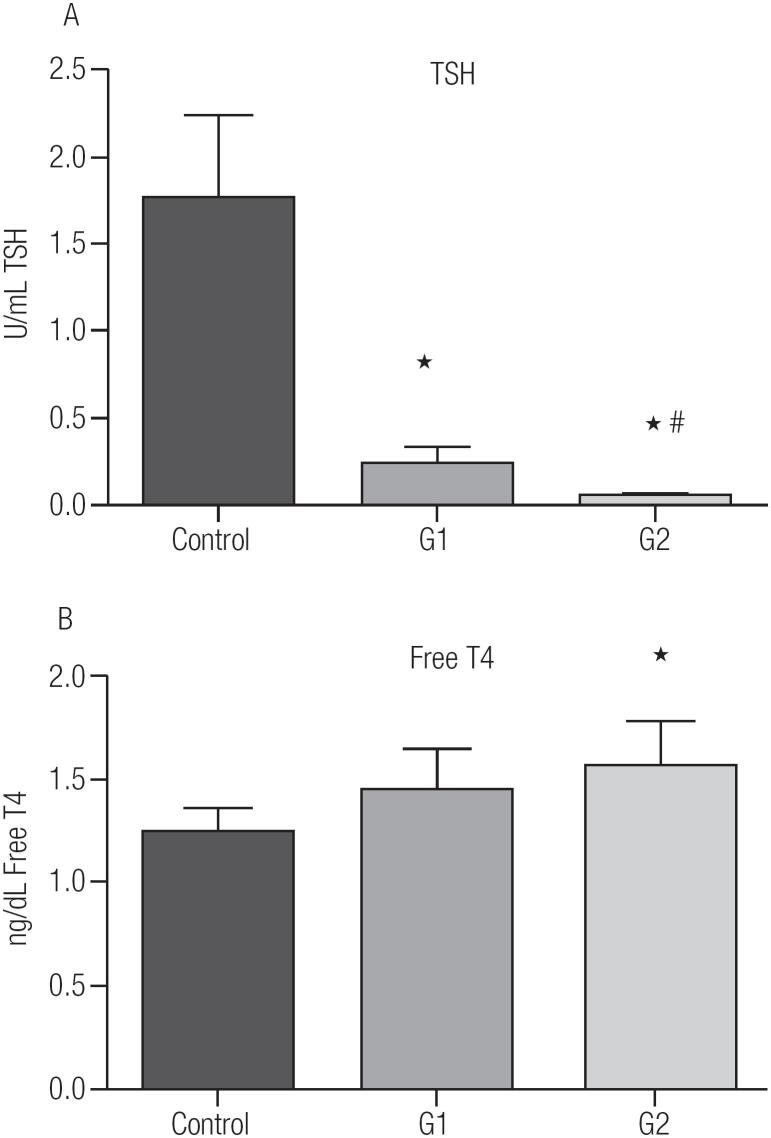
Hormonal concentration of (**A**) Thyroid stimulating hormone (TSH) and (**B**) Free thyroxine FT4. Control group - euthyroid (6); G1-TSH ≥ 0.1 to 0.4 UI/mL (n = 21); G2-TSH < 0.1 UI/mL (n = 25). Data are presented as mean ± SD. * p < 0.05 vs. control; ^#^ p < 0.05 vs. G1.

Table 1 shows the general characteristics of the participants grouped according to their TSH suppression levels. There were no statistical differences between the groups in terms of age, height or weight. Figure 1A shows that control group have regular TSH levels that are higher than G1 or G2. However, G2 was more suppressed than G1 (TSH = 0.03 ± 0.22 IU/mL vs. TSH = 0.005 ± 0.022 IU/mL, p < 0.001, respectively). The FT4 concentration was similar in both groups (G1 and G2) with the FT4 levels in G2 being higher than the control group (Figure 1B).

**Table 1:** Sample characteristics

	Control	G1 (TSH ≥ 0.1 to 0.4 IU/mL)	G2 (TSH < 0.1 IU/mL)
Age (years)	52.2 ± 7.1	46 ± 16	55 ± 11
Weight (kg)	67 ± 9.5	78 ± 14.2	75.9 ± 16.4
Height (m)	1.7 ± 0.1	1.7 ± 0.1	1.6 ± 0.1
Gender	3 women; 3 men	14 women; 7 men	21 women 4 men

Control group – euthyroid (6); G1-TSH ≥ 0.1 to 0.4 UI/mL (n = 21); G2-TSH < 0.1 UI/mL (n = 25). Data are presented as mean ± SD.

**Figure 2:**
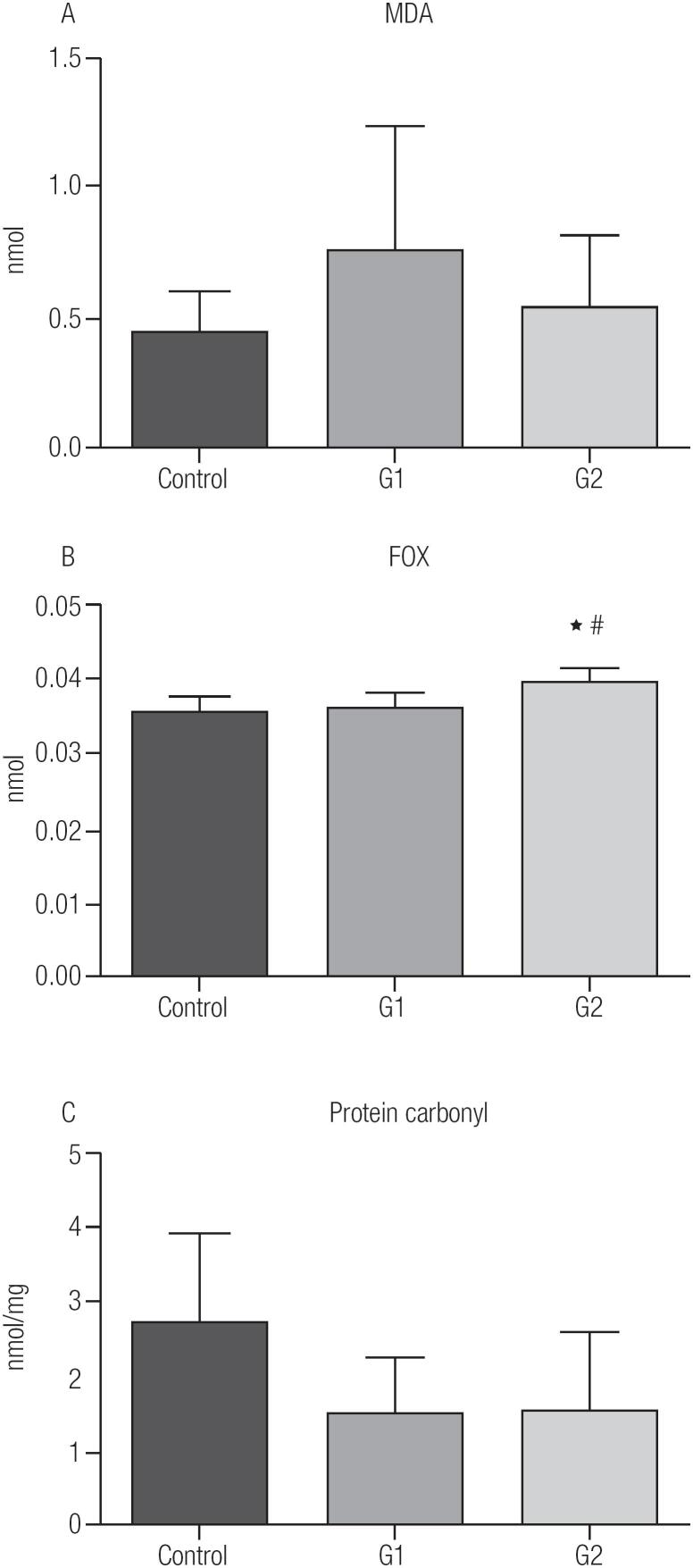
Lipid peroxidation (MDA - malondialdehyde) (**A**), FOX (**B**), protein carbonyl (**C**). Control group - euthyroid (6); G1-TSH ≥ 0.1 to 0.4 UI/mL (n = 21); G2-TSH < 0.1 UI/mL (n = 25). Data are presented as mean ± SD. *p < 0.05 vs. control; #p < 0.05 vs. G1.

The level of lipid peroxidation as measured by TBARS was similar in all three groups (Figure 2A). In contrast, when the levels were measured by the FOX assay, G2 showed an increase in lipid peroxidation in comparison to the control and G1 (Figure 2B). While the lipid peroxidation levels in G2 suggested oxidative damage, the evaluation of the extent of protein carbonylation showed no significant differences between the three groups (Figure 2C).

Both of the SCH groups (G1 and G2) did present higher values than the control group in the plasma concentration of all cell damage markers (Table 2). In the group comparisons, G1 appeared to show greater significant differences than G2 to controls for AST (control vs G1, p = 0.0003; control vs G2, p = 0.001); ALT (control vs G1, p = 0.0008; control vs G2, p = 0.01), GGT (control vs G1, p = 0.02; control vs G2, p = 0.03), and CK (control vs G1, p = 0.01; control vs G2, p = 0.04).

**Table 2:** Plasma biomarkers of cellular damage

	Control	G1 (TSH ≥ 0.1 to 0.4 IU/mL)	G2 (TSH < 0.1 IU/mL)
AST (U/L)	10.51 ± 1.33	22.19 ± 0.99*	22.36 ± 0.94*
ALT (U/L)	27.03 ± 3.58	40.53 ± 2.47*	43.32 ± 2.41*
GGT (U/L)	17.83 ± 3.3	42.29 ± 5.08*	42.60 ± 4.52*
CK (U/L)	65.44 ± 30.48	135.9 ± 9.32*	143.1 ± 19.43*

AST: aspartate aminotransferase; ALT: alanine aminotransferase; GGT: gamma glutamine transferase; CK: creatine kinase. Control group - euthyroid (6); G1-TSH ≥ 0.1 to 0.4 UI/mL (n = 21); G2-TSH < 0.1 UI/mL (n = 25). Data are presented as mean ± SD.

* p < 0.05 vs. control; p < 0.05 vs. G1.

**Figure 3:**
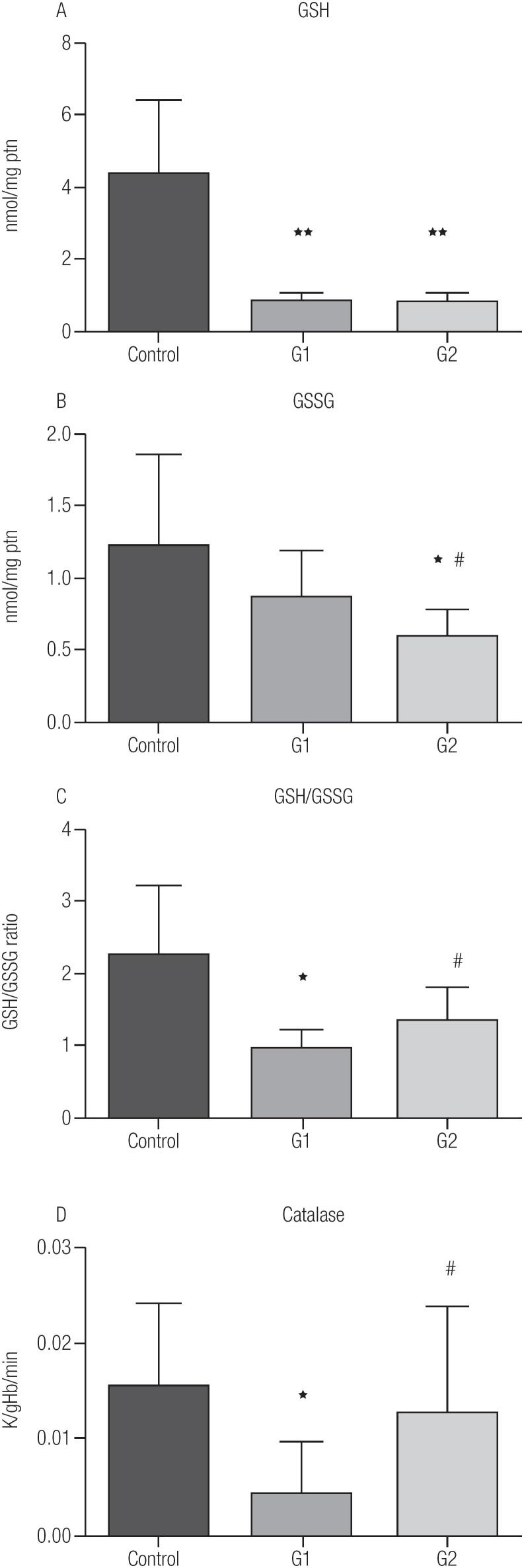
Antioxidant profile: (**A**) GSH; (**B**) GSSG; (**C**) GSH/GSSG and (**D**) Catalase. G1-TSH ≥ 0.1 to 0.4 UI/mL (n = 21); G2-TSH < 0.1 UI/mL (n = 25). GSH, reduced glutathione; GSSG, oxidized glutathione; GSH/ GSSG, ratio reduced glutathione/oxidized glutathione. Control group – euthyroid (6); G1-TSH ≥ 0.1 to 0.4 UI/mL (n = 21); G2-TSH < 0.1 UI/mL (n = 25). Data are presented as mean ± SD. *p < 0.05 vs. control; ^#^p < 0.05 vs. G1.

Figure 3A shows that the GSH concentration was higher in both suppressed groups. For GSSG, its concentration was lower in G2 when compared with G1 and control group (Figure 3B). To estimate the redox balance of these patients, GSH/GSSG ratio was calculated (Figure 3C). The GSH/GSSG ratio was smaller in G1 than control (p = 0.0053) with the ratio of G2 being greater than G1. The catalase activity (Figure 3D) was lower in G1 than control and G2 (p = 0.0006 and 0.03, respectively).

## DISCUSSION

There is a consensus in the literature that hyperthyroidism induces lipid peroxidation (5,12,13), which is hallmark of oxidative stress. Yavuz and cols. (8) showed that SCH promotes oxidative stress that was associated with endothelial impairment. However, the study did not relate the level of oxidative stress to different TSH levels. To date, no research has evaluated the level of lipid peroxidation among patients with regards to different levels of suppression. The aim of the study reported here was to compare the redox profiles of patients with exogenous SCH considering two categories of TSH suppression: ≥ 0.1 to 0.4 IU/mL (G1) and < 0.1 IU/L levels of TSH suppression in exogenous SCH (4).

Our measurements of lipid peroxidation using the FOX assay showed higher levels associated with the exogenous subclinical hyperthyroid state in the G2 group compared with the less suppressed (G1) and control group (Figure 2B). In contrast, measurements by TBARS did not show any differences between the groups. While both the FOX and TBARS assays measure lipid peroxidation, these methods have different affinities for the intermediaries of the lipid peroxidation cascade. As such, it is not uncommon for their results to show different behaviors (17), which suggests that G2 group did experience oxidative stress.

Another oxidative marker is carbonyl groups on proteins (23). Here, the SCH groups did not show any difference when compared with control (Figure 2C). This was unexpected since Mseddi and cols. (24) had observed that patients with Hashimoto's thyroiditis had increased plasma protein carbonyl. Considering that Hashimoto's thyroiditis is associated with an increased inflammatory profile and oxidative stress (25) while the SCH patients here had no active inflammatory process, the presence or absence of differences in protein carbonyl levels can be associated with the type of disease. Since our patients have exogenous subclinical hypertodism, the results suggest that exogenous hormonal variation alone could not significantly increase protein carbonyl levels.

Elevated T4 levels, together with free radical production, has been associated with hepatic cellular damage as observed by transaminases in the plasma (26). Another study in rats also showed also that an excess of T4 increased liver injury as measured by increases in AST, ALT, and GGT plasma concentration in rats (27). The results from the G2 group, which have higher T4 concentrations in comparison to the control group (Figure 1B), are consistent with these previous reports with increases in AST, ALT and GGT (Table 2). However, the G1 group, which has T4 levels within the normal range (Figure 1B), also presented with an increase in the levels of the biomarkers for cell lesion (Table 2). In this case, the increased in these biomarkers of hepatic lesion can be associated to the reduction of redox equilibrium observed in this group (Figure 3C), also in agreement with Massarah and Boumendjal (26). It is interesting to observe that both AST and GGT were higher than the normal reference values in G1 and G2 (normal values: AST < 32 U/L, GGT < 38 U/L), indicating hepatic damage induced by TSH suppression. As well as the hepatic biomarkers of cell damage, we also observed an increased plasma concentration of CK, which is characteristic for muscular damage (28). These measured increases in circulating enzyme levels in patients with TSH suppression suggest an affect not only on hepatic tissue, but also muscle.

Defensive measures to prevent cellular damage from oxidative processes include increased antioxidants either through enzymatic or non-enzymatic reactions. The major endogenous non-enzymatic antioxidant is glutathione, which can be in its reduced (GSH) or oxidized (GSSG) form and the ratio of GSH to GSSG provides indications of the tissue redox balance (29). In the G1 and G2 groups, the reduced glutathione (GSH) level, antioxidant was lower when compared with the control group (Figure 3A), which suggests a reduced antioxidant capacity in both TSH suppressed groups. For the oxidized form (GSSG), G2 had a lower concentration compared to G1 and control group (Figure 3B). The redox equilibrium calculated from the GSH/GSSG ratio was diminished in G1 compared to the control and G2 groups (Figure 3C). The reduced imbalance in redox equilibrium observed for the G2 group is most likely a result of the increase in catalase activities (Figure 3D). In a rat model of hyperthyroidism, catalase liver activity, GPx, and lipid peroxidation were all increased suggesting that increased oxidative damage, as observed in G2, can induce increases of antioxidants enzymes activity to control the imbalance (26). This is consistent with our results for G2, since it was the only group that presented with an increase in lipid peroxidation (Figure 2B) and it was the most TSH suppressed group (G2).

This study is the first attempt to understand redox status and possible cellular damage from oxidative stress in SCH patients presenting with TSH at different levels. The results suggest that TSH suppression therapy with LT4 that leads to subclinical hyperthyroidism can cause different oxidative responses when patients are segregated by their TSH suppression levels into two groups (TSH < 0.01 IU/mL; TSH between 0.1 to 0.4 IU/mL). Both groups showed hepatic damage, but the group with lower TSH (G2) displayed higher levels of lipid peroxidation as well as increased antioxidant capacity. In conclusion, we encourage subclinical hyperthyroidism researchers to categorize patients by their TSH levels as suggested by Biondi and cols. 2015, since different oxidative damage can aggravate or attenuate diseases associated with subclinical hyperthyroidism. Future studies will help understand how these different interfere on physiological functions.
